# Targeting Apolipoprotein E/Amyloid β Binding by Peptoid CPO_Aβ17-21 P Ameliorates Alzheimer’s Disease Related Pathology and Cognitive Decline

**DOI:** 10.1038/s41598-017-08604-8

**Published:** 2017-08-14

**Authors:** Shan Liu, Shinae Park, Grant Allington, Frances Prelli, Yanjie Sun, Mitchell Martá-Ariza, Henrieta Scholtzova, Goutam Biswas, Bernard Brown, Philip B. Verghese, Pankaj D. Mehta, Yong-Uk Kwon, Thomas Wisniewski

**Affiliations:** 1 Center for Cognitive Neurology, Department of Neurology, New York University School of Medicine, New York, USA; 2 Department of Chemistry and Nanoscience, Ewha Womans University, Seoul, Korea; 3 Department of Chemistry, New York University, New York, USA; 4 C2N Diagnostics, Center for Emerging Technologies, 4041 Forest Park Avenue, St. Louis, MO 63108 USA; 5 Department of Immunology, New York State Institute for Basic Research in Developmental Disabilities, New York, USA; 6 Center for Cognitive Neurology, Departments of Neurology, Psychiatry and Pathology, Neuroscience Institute, New York University School of Medicine, New York, USA

## Abstract

Inheritance of the apolipoprotein E4 (apoE4) genotype has been identified as the major genetic risk factor for late onset Alzheimer’s disease (AD). Studies have shown that apoE, apoE4 in particular, binds to amyloid-*β* (A*β*) peptides at residues 12-28 of A*β* and this binding modulates A*β* accumulation and disease progression. We have previously shown in several AD transgenic mice lines that blocking the apoE/A*β* interaction with A*β*12-28 P reduced A*β* and tau-related pathology, leading to cognitive improvements in treated AD mice. Recently, we have designed a small peptoid library derived from the A*β*12-28 P sequence to screen for new apoE/A*β* binding inhibitors with higher efficacy and safety. Peptoids are better drug candidates than peptides due to their inherently more favorable pharmacokinetic properties. One of the lead peptoid compounds, CPO_A*β*17–21 P, diminished the apoE/A*β* interaction and attenuated the apoE4 pro-fibrillogenic effects on A*β* aggregation *in vitro* as well as apoE4 potentiation of A*β* cytotoxicity. CPO_A*β*17–21 P reduced A*β*-related pathology coupled with cognitive improvements in an AD APP/PS1 transgenic mouse model. Our study suggests the non-toxic, non-fibrillogenic peptoid CPO_A*β*17–21 P has significant promise as a new AD therapeutic agent which targets the A*β* related apoE pathway, with improved efficacy and pharmacokinetic properties.

## Introduction

The pathological accumulation of A*β* peptides as toxic oligomers, amyloid plaques and cerebral amyloid angiopathy (CAA), either from increased production of A*β* peptides or from their inadequate clearance, is critical in the pathogenesis of Alzheimer’s disease (AD)^1, 2^. The apolipoprotein E4 (apoE4) allele, a major genetic risk factor for late-onset AD, has been strongly associated with increased amyloid plaques deposition in brain parenchyma and advanced vascular amyloid pathology such as CAA^3–5^. Numerous studies have shown that apoE binds to residues 12–28 of A*β* and this binding modulates A*β* accumulation hence affecting disease progression^3, 4, 6–13^. However, there is no consensus on how different apoE genotypes contribute to the pathogenesis of AD^3, 6, 7^. It has been hypothesized that apoE and apoE4 in particular is an amyloid catalyst or “pathological chaperone”^3, 8^ and that the interaction of Aβ and apoE4 is associated with neurotoxicity^14^. Alternatively it has been posited that apoE is an A*β* clearance factor, with apoE4 being impaired at this function compared to apoE3 or E2^6, 15^. We suggest that the optimal therapeutic strategy may be to specifically prevent the interaction of apoE with A*β*, allowing to circumvent complications of altering apoE levels which may cause detrimental effects on the many other beneficial roles apoE plays in neurobiology^3, 7^.

We and others have previously shown that blocking the apoE/A*β* interaction by A*β*12-28 P peptides could constitute a novel treatment for AD by reducing brain parenchymal and vascular amyloid burden as well as tau related pathology in several AD transgenic mice lines^16–20^. Recently, using peptidomimetic technology and the macrocyclization method, we designed a peptoid library derived from A*β*12-28 P sequence to screen for new apoE/A*β* binding inhibitors with higher efficacy and safety for blocking the apoE/A*β* interaction^21–23^.

Peptoids are N-substituted glycine oligomers, which recapitulate many desirable attributes of natural peptides including formation of stable secondary structures and demonstration of a range of biological activities^24, 25^. Advantages of peptoids over peptides include stability of their structure to excessive temperature (over 75 °C), salt, pH, and organic solvent. They are resistant to proteolytic degradation and can be excreted whole in urine, which is an important attribute of a pharmacologically useful peptide mimic^24, 25^. Furthermore, peptoids lack the hydrogen of secondary amides, and thus are unlikely to form the *β*-sheet structure, which is associated with the toxicity of A*β* and A*β* derived peptides. Peptoids can be easily synthesized by solid phase submonomer synthesis using hundreds of available primary amines and modified by various chemical ligation methods to enhance the desired biological function and/or properties. A number of peptoid based compounds have been successfully developed and introduced to clinical practice^24, 25^. The design of biologically-active peptoid sequences of some of these compounds was achieved by the systematic modification of an active peptide target sequence, which is the same strategy we are using to design our peptoid library for screening.

In this study, we report the synthesis of a library of peptoids based on the A*β*12-28 P sequence. We screened this library for the ability of the peptoids to diminish apoE/A*β* binding. In our report we tested the ability of CPO_A*β*17-21 P, one of the lead peptoids obtained from the inhibition potency screening, to diminish the apoE/A*β* binding *in vitro* monitored by surface plasmon resonance (SPR). We also tested its ability to inhibit the promoting effects of recombinant apoE4 on A*β*40 and A*β*42 aggregation using a Thioflavin T assay. We have tested the potential toxicity of CPO_A*β*17-21 P using mouse N2a and human SK-N-SH neuroblastoma cell lines. We also assayed if CPO_A*β*17-21 P could inhibit the potentiation of apoE4 on the cytotoxicity of A*β*42 using a human SK-N-SH neuroblastoma cell line. Furthermore, we found that *in vivo* administration of CPO A*β*17-21 P in an APP/PS1transgenic mouse AD model could reduce pathology and produce cognitive benefits.

## Materials and Methods

### Materials and regents

Unless stated otherwise all chemicals and regents were purchased from Sigma-Aldrich (St. Louis, MO). The A*β*12-28 P, A*β*40 and A*β*42 peptides were synthesized and purified at ERI Amyloid Laboratory LLC (Oxford, CT) as previously described^19^. For SPR binding assay, aggregation studies and oligomeric A*β*42 cytotoxicity assessment, the A*β* peptides were treated with 1, 1, 1, 3, 3, 3-hexafluoro-2-propanol (HFIP) to remove secondary structure and render peptides into monomeric forms^26^. The CPO_A*β*17-21 P and other peptoid compounds were designed and synthesized in the laboratory of Prof. Yong-Uk Kwon at Ewha Womans University (Korea). Recombinant human apoE4 was purchased from BioVision (Milpitas, CA) and reconstituted in ddH_2_O to a concentration of 1 mg/ml. Human lipidated apoE3 and apoE4 were prepared as previously published^15^. All cell lines were purchased from American Type Culture Collection (ATCC, Manassas, VA) and kept in Dulbecco’s modified Eagle’s medium (DMEM) with 10% fetal bovine serum (FBS), 100 units penicillin and 100 µg streptomycin/mL. All cell culture regents were purchased from Thermo Fisher Scientific (Waltham, MA).

### Library design and synthesis of peptoids

Our library design is a systematic intelligent design approach. A series of linear and cyclic peptoids derived from A*β*12-28 were designed and synthesized on solid-phase. The sequential steps in peptoid compound synthesis involved: 1) abbreviation of A*β*12-28 sequence to the core sequence A*β*15-24 capable of blocking the apoE/A*β* interaction; 2) substitution of amino acids in shortened peptides for non-natural “peptoid” residues; 3) construction of cyclic peptoids using macrolactamization. This is a modification which is known to increase the stability of peptoids, improve cell/blood-brain barrier (BBB) permeability and typically increase the affinity of binding^21, 22, 25^. Peptoid synthesis was done using methods as previously described^23, 27^. Briefly, peptoids were easily prepared on solid-phase by the submonomer strategy employing bromoacetic acid and primary amines. The polar side chain-containing amines were readily introduced under the modified synthetic conditions. For the construction of cyclic peptoids, the macrolactamization between the carboxylic acid functionality of β-alanine unit and the amino functionality of the terminal peptoid unit was successfully carried out in the presence of the coupling agent PyAOP^23, 27^.

### SPR monitoring

Experiments were performed using a Reichert SR7000DC refractometer system (Reichert, New York) equipped with a CM5 sensor chip. Acetate buffer was used for immobilization of the peptide A*β*42 (ligand). We used Aβ42 for these binding studies (and not Aβ40), since Aβ42 species are the major constituents of amyloid plaques, with Aβ40 species being more common in vascular amyloid^28, 29^; although some reports suggest that Aβ40 species overall can be the most abundant in the AD cortex^30, 31^. Importantly, Aβ42 species are considered critical for seeding of amyloid deposits^2, 32^. CM-dextran on a CM5 gold sensor chip was activated by injecting a solution of 10 mg/mL N-Hydroxysuccinimide (NHS) and 40 mg/mL 1-Ethyl-3-(3-dimethylaminopropyl) carbodiimide (EDC) over the sensor chip surface for 7 min at a flow rate of 20 µL/min. Then the ligand, 50 µg/mL HFIP treated synthetic peptide A*β*42 in 10 mM sodium acetate, pH 4.5, was immobilized by injection over the surface for 20 min. The unreacted sites on the sensor chip surface were blocked by injection of 1 M ethanolamine, pH 8.5 for 10 min. Analytes containing recombinant human apoE4 molecules pre-incubated with various peptoid inhibitors at different concentrations in phosphate buffered saline with 0.05% TWEEN 20, pH 7.4 (PBST) were injected over the surface and the association and dissociation reactions were monitored. After each binding cycle the surface was regenerated by a short, fast injection of 10 mM hydrochloric acid. To verify results analytes containing lipidated human apoE4 molecules or lipidated human apoE3 molecules incubated with peptoid inhibitors in phosphate buffered saline with 0.05% TWEEN 20, pH 7.4 (PBST) were tested and the association and dissociation reactions were monitored.

### Aggregation assay

Peptide A*β*42 or peptoid CPO_A*β*17-21 P were incubated alone at a concentration of 100 µM over a period of 7–10 days at 37 °C in 100 mM Tris buffer (pH 7.4). A*β*42 was also incubated in the presence of 1 µM of recombinant human apoE4 or CPO_A*β*17–21 P (100:1 molar ratio), under the same conditions. In aggregation inhibition experiments, apoE4 was pre-incubated with CPO_A*β*17-21 P in a molar ratio of 1:2 for 6 hrs at 37 °C followed by addition to freshly reconstituted A*β*42 on ice (molar ratio 100:1:2, A*β*42:apoE4:peptoid). As a control, A*β*42 was also incubated with CPO_A*β*17-21 P at a molar ratio of 100:2. For peptide A*β*40 all the experiments were conducted under the same conditions as A*β*42 except the peptide concentration was 400 µM. The molar ratio of A*β*40:apoE4:CPO_A*β*17-21 P was 100:1:2. (400 µM A*β*40:4 µM apoE4:8 µM CPO_A*β*17-21 P). Fibril formation by the different peptides at various time points was evaluated by a standard Thioflavin-T assay according to previously published protocols^13, 33^.The samples were diluted in buffer containing 50 mM glycine, 2 µM Thioflavin T pH 9.2, and read on a Molecular Devices SpectraMax M2 spectrophotometer (Sunnyvale, CA) at excitation and emission wavelengths 440 and 482 nm.

### Cell viability assay

The effects of 0.1 to 10 µM concentrations of CPO_A*β*17-21 P on the viability of SK-N-SH human or N2a mouse neuroblastoma cell line were assessed initially for peptoid compound cytotoxicity in cell cultures. Cells from the fifth passage and beyond were seeded in fifty-eight of the central sixty wells of a Greiner 96 well cell culture plate (Baton Rouge, LA) with clear flat bottom and black wall at 5,000 cells per well leaving two of the central wells unseeded as blank control. The next day, cells were washed twice carefully with warm plain medium and different concentrations of CPO_ A*β*17-21 P diluted in plain DMEM medium containing 1% N-2 supplement and added to cells at a final volume of 100 μl per well. Every treatment group contained 5-6 replicates. 100 µL of distilled water was added to the side wells and corner wells of the plate in order to minimize evaporation during incubation. The plate was incubated at 37 °C 5% CO_2_ for 24 hrs followed by cell viability assay using Promega CellTiter-Blue® Cell Viability Assay kit (Madison, WI) according to the product manufacturer’s instructions. Briefly, 20 µL of CellTiter-Blue regent was added to each of the central 60 wells and the plates were incubated at 37 °C 5% CO_2_ for 1–4 hrs to allow live cells to convert a redox dye resazurin into a fluorescent end product resorufin, and the fluorescent signal was measured by a Synergy H-1 Hybrid Reader (BioTek Instruments, Winooski, VT) at 560_Ex_/590_Em._ All experiments were run in triplicate.

In toxicity rescue experiments CPO_A*β*17–21 P or A*β*12–28 P (as positive control) were used to neutralize recombinant human apoE4’s effects on oligomeric A*β*42 neurotoxicity. Oligomeric A*β*42 was prepared as previously published^26^. To prepare 100 µM oligomeric A*β*42 stock solution, 0.45 mg HFIP pretreated A*β*42 peptide was dissolved into 20 µl fresh dry dimethyl sulfoxide (DMSO) to form 5 mM A*β*42 DMSO solution; then 980 µl ice cold phenol-free Ham’s F-12 cell culture medium (with 146 mg/L L-Glutamine) was added to the Aβ42 DMSO solution followed by incubation at 4 °C for 24 hrs. CPO_ A*β*17–21 P or A*β*12-28 P was pre-incubated with apoE4 in DMEM medium containing 1% N-2 supplement for 6 hrs at 37 °C then mixed with freshly prepared oligomeric A*β*42 and added to SK-N-SH cells. For comparison, cells were incubated with A*β*42 alone and A*β*42 + apoE4, apoE alone, A*β*42 + CPO_A*β*17-21 P and A*β*42 + A*β*12-28 P as additional controls. The final concentration of peptides or peptoids was as follows: A*β*42 10 µM, apoE4 1 µM, A*β*12-28 P 4 µM and CPO_A*β*17-21 P 1 µM or 4 µM. Cells were under different treatments for three days and the cell viability was assessed as described above.

### Transgenic mice and treatment

The effect of CPO_A*β*17-21 P on A*β* deposition was tested in APP/PS1 Tg mice with plaque pathology^34^. This AD mouse model develops plaque pathology from the age of 3 months. Animals were divided into two groups and received intraperitoneally (i.p.) injections of 0.2 mg of CPO_ A*β*17–21 P per mouse in sterile saline (n = 11, 5 male, 6 female) or saline alone (n = 10, 5 male, 5 female) as a control twice weekly for 4 months, beginning at age 3 months. During the treatment, veterinary staff monitored animals for any signs of toxicity, such as changes in body weight, physical appearance, and altered behavior. Animals were sacrificed a week after administration of the last treatment dose and behavioral testing. All mouse care and experimental procedures were compliant with guidelines of animal experimentation and were approved by the Institutional Animal Care and Use Committee at the New York University School of Medicine.

### Behavioral testing

Behavioral tests were performed during the last month of treatment. All testing were conducted by an individual blinded to the treatment assignment. To verify that any treatment effects observed in the cognitive tests are not due to differences in motor abilities, sensorimotor tests including locomotor activity, rotarod and traverse beam were performed to measure general motor functions, coordination and balance, as previously described^35–37^. Radial arm maze test and novel object recognition test were performed to evaluate treatment associated cognitive improvements of spatial learning (working memory) and short-term memory as previously described^19, 35, 37^.

#### Locomotor Activity

Mouse locomotor activity was recorded by a Hamilton-Kinder Smart-frame Photobeam System (Kinder Scientific, Poway, CA). Following habituation in a circular open field chamber (70 × 70 cm) for 15 min, each mouse was allowed to explore the environment for 15 min. Horizontal movements of the mice were automatically recorded and results were reported as distance traveled (cm), average and maximum travel velocity (cm/s) and mean resting time (s) of the mouse.

#### Rotarod

Mice were habituated to reach a baseline level of performance, before being tested for 5 min on a 3.6 cm diameter rod (Rotarod 7650 accelerating model; Ugo Basile Biological Research Apparatus, Varese, Italy) with initial speed set at 1.5 rpm then raised every 30 sec by 0.5 rpm. To access performance, the speed of the rod was recorded when the mouse fell or inverted (by clinging) from the top of the rotating barrel during three test trials. A soft cushion was placed under the rod to prevent injury from falling.

#### Traverse Beam

Mice were assessed by their ability to traverse a graded narrow wooden beam to reach a goal box. During the test, mice were placed on a 1.1 cm wide beam, 50.8 cm long, 30 cm above a padded surface, in a perpendicular orientation to habituate, and were then monitored for a maximum of 60 sec. The number of foot slips each mouse made before falling or reaching the goal box was recorded for each of three successive trials. The average numbers of foot slips of the mice from different treatment groups in three trials were calculated.

#### Radial Arm Maze

Spatial learning (working memory) was tested using a radial arm maze with eight radial 30-cm-long arms originating from the central space. A well baited with 0.2 ml of 0.1% saccharin solution was placed at the end of each arm. Mice were deprived of water for 24 hrs before testing, and then water access was restricted to 1 hr per day for the duration of testing. The task required an animal to enter all arms and drink the saccharin solution until the eight rewards had been consumed. After 3 days of adaptation, mice were subjected to testing for 9 consecutive days. The number of errors (entry into previously visited arms) was recorded during each session.

#### Object Recognition

The spontaneous object recognition test measures short-term memory. It was conducted in a square-shaped open-field box (48 cm square, with 18 cm-high walls constructed from black Plexiglas). Objects were placed diagonally in the center of two zones and mice were allowed to explore two identical objects in the open field for 15 min. 24 hrs later this procedure was repeated with two novel identical objects. At the end of the training phase, mice were removed from the box for the duration of the retention delay (3 hrs). During retention session, one of the previous familiar objects used during training was replaced by a second novel object and the animal was allowed to explore freely for 6 min. The amount of time spent exploring each object within a defined zone of the area was automatically recorded by a tracking system (San Diego Instruments, San Diego, CA).

### Tissue processing and immunohistochemistry

Mice were anesthetized with sodium pentobarbital (150 mg/kg, i.p.) and blood was collected via cardiac puncture and plasma was separated by centrifugation. Animals were perfused transaortically. The brains and internal organs were collected and cut into halves, one half immersion-fixed in periodate-lysine paraformaldehyde (PLP) for histopathological analysis, and another half snap-frozen for biochemical studies as previously described^19^. Serial coronal brain sections (40 μm) were cut evenly spaced every 400 μm μm along the entire rostro-caudal brain axis and stained for immunohistochemical analysis with: (1) a mixture of anti-A*β* monoclonal antibodies 6E10/4G8 (1 :2000) (Covance, Princeton, NJ), (2) polyclonal anti-glial fibrillary acidic protein (GFAP) antibody (1:1000) (Dako, Carpinteria, CA), (3) polyclonal anti-Iba1 antibody (1:1000) (Wako Chemicals, Richmond, VA) and (4) monoclonal anti-CD11b (1: 500) (Serotec, Raleigh, NC). To measure treated mice brain total amyloid burden change, two different anti-A*β* monoclonal antibodies with distinct epitopes 6E10 (epitope A*β*1–16) and 4G8 (epitope A*β*17–24) were used^38^. To examine brain inflammation levels associated with treatment, we assessed the degree of astrocytosis with GFAP immunoreactivity and the degree of microgliosis with Iba1 and CD11b immunoreactivity as previously published^19, 37, 39, 40^. GFAP is a component of the glial intermediate filaments that form part of the cytoskeleton and is found predominantly in astrocytes^36^. CD11b is a protein subunit that forms integrin alpha-M beta-2 (αMβ2) molecule, also known as MAC-1 or complement receptor 3 (CR3). CD11b monoclonal antibodies are commonly used for detection of microglial activation at the earliest stages of plaque development^41^. In addition, Iba1 (ionized calcium binding adaptor molecule) antibody was used to label the entire microglia population including activated and resting microglia, a marker for detecting generic microglia^42, 43^. Immunohistochemistry was performed on free floating coronal brain sections. Details of the procedure and techniques were described previously^19, 37, 39, 40^. Antibody staining was revealed with 3, 3’-diaminobenzidine (DAB; Sigma-Aldrich) and nickel ammonium sulfate (Ni; Mallinckrodt, Paris, KY) for intensification. Human Alzheimer disease patients brain sections were used for positive control immunostaining, sequential mouse brain sections with omission of the primary antibody were used for negative immunostaining controls.

### Quantification of amyloid burden, astrocytosis and microgliosis

Amyloid burden was measured by a Bioquant stereology (BIOQUANT Image Analysis Corporation, Nashville, TN) semi-automated image analysis system using a randomized and unbiased sampling scheme according to our previous published protocol^17, 19, 37, 39, 40^. The total amyloid burden (defined as the percentage of test area occupied by A*β* immunoreactivity) was quantified for the cortex and hippocampus on coronal plane sections stained with a mixture of anti-A*β* antibodies 6E10 (epitope A*β*1–16)/4G8 (epitope A*β*17–24). The staining resulted in black A*β* immunoreactivity due to the intensification with nickel ammonium sulfate and facilitated threshold detection. The cortical area was measured as dorsomedial from the cingulate cortex and extended ventrolaterally to the rhinal fissure within the right hemisphere. Test areas (640 µm × 480 µm) were randomly selected by a grid (800 µm × 800 µm) over the traced contour. 8 sections, approximately 80 areas, were analyzed per animal. Hippocampal measurements (grid area, 600 µm × 600 µm) were performed similar to the cortical analysis (7 sections, and approximately 85 areas per animal).

The assessment of astrocytosis and microgliosis were based on a semi-quantitative analysis, using methods we have previously published by a rater blinded to the treatment status of the mice^19, 37, 39, 40^. Prior to analysis, brains were microscopically examined and rated on a scale from 0–4, in increments of 0.5, depending on the degree of pathology and/or the activation stage of the glial cells. All stained cortical and hippocampal sections were analyzed at 10x magnification. Approximately 8 cortical sections and 6 hippocampal sections were analyzed per animal. Astrogliosis evaluation was based on GFAP immunoreactivity (number of GFAP immunoreactive cells and complexity of astrocytic branching) on a scale from 0 (few resting astrocytes) to 4 (numerous reactive astrocytes/extensive branching), as previously published^19, 37, 39, 40^. Microgliosis was assessed on CD11b and Iba1 immunostained sections with rating from 0 (few resting microglia) to 4 (numerous ramified/phagocytic microglia) in increments of 0.5.

### Assessment of levels of A*β* and oligomeric A*β* in the brain by ELISA

Brains were weighed and homogenized (10% w/v) in tissue homogenization buffer (20 mM Tris base, pH 7.4, 250 mM sucrose, 1 mM EDTA, 1 mM EGTA) with 100 mM phenylmethylsulphonyl fluoride, complete protease inhibitor cocktail and PhosSTOP phosphatase inhibitor cocktail (Roche, Indianapolis, IN, USA) added immediately before homogenization. The extraction of soluble A*β* and total A*β* was according to the method we have published previously^37, 44^. Brain homogenates were mixed with an equal volume of cold 0.4% diethylamine (DEA)/100 mM NaCl, and subsequently centrifuged at 100,000 × g for 1 hr at 4 °C. The supernatant was neutralized with 1/10 volume of 0.5 M Tris, pH 6.8, flash-frozen on dry ice and stored at −80 °C until used for ELISA analysis as soluble A*β* fraction. For extraction of the total A*β*, homogenates (200 μl) were added to 440 μl of cold formic acid (FA) and sonicated for 1 min on ice. Subsequently, 400 μl of this solution was centrifuged at 100,000 × g for 1 h at 4 °C and 210 μl of the resulting supernatant was diluted into 4 ml of FA neutralization solution (1 M Tris base, 0.5 M Na_2_HPO_4_, 0.05% NaN_3_), flash-frozen on dry ice and stored at −80 °C until used for total A*β* measurement. Soluble and total A*β* levels in the brain were measured by sandwich ELISA which used 6E10 monoclonal antibody as a capture antibody and two detection polyclonal antibodies R162 and R165 specific for A*β*40 and A*β*42, respectively. The assay was performed by an investigator (Pankaj Mehta) who was blinded to treatment group assignment. The levels of A*β* species are presented as μg of A*β* per g of wet brain, taking into account the dilution during brain homogenization and extraction procedures.

Oligomeric A*β* (o-A*β*) levels were determined with the Biosensis Oligomeric Amyloid-Beta ELISA kit (Biosensis, Thebarton, South Australia) following the manufacturer’s instructions. In brief, the unknown concentrations of o-A*β* in samples were measured against a standard containing different concentrations of o-A*β*. Samples diluted in the provided diluent buffer were incubated for 24 hrs at 2–8 °C allowing the A*β* to bind to the pre-coated capture antibody (MOAB-2), followed by extensive washing and incubation for 1 hr at RT with biotin conjugated detection antibody (same as the capture antibody) which binds to the immobilized o-A*β*. After removal of excess antibody, horseradish peroxidase (HRP)-conjugated streptavidin (SAV-HRP) was added to incubate for 30 min, followed by washing, and a tetramethylbenzidine (TMB) substrate incubation which resulted in a colorimetric solution. The intensity of this colored product is directly proportional to the concentration of o-A*β* in the sample. The TMB reaction was stopped and the absorbance of each well was read at 450 nm. The standards provided a linear curve and the best-fit line determined by linear regression were used to calculate the concentration of A*β* oligomers in the samples

### Western blot analysis of A*β* oligomers

Brain homogenates were centrifuged at 100,000 × g for 1 hr at 4 °C. The supernatants were collected and the total protein concentrations were determined by the bicinchoninic acid assay (BCA; Pierce, Rockford, IL). Equal aliquots of protein from each sample, mixed with an equal volume of Tricine sample buffer, were subjected to overnight electrophoresis on 12.5% sodium dodecyl sulfate polyacrylamide Tris-tricine gels under non-reducing conditions. Following overnight electrophoresis proteins were transferred onto nitrocellulose membranes (BioRad, CA) for 1 h at 400 mA using CAPS buffer (3-cyclohexylamino-1-propanesulfonic acid) containing 10% methanol. The membranes were blocked with 5% non-fat milk in TBST (Tris, 10 mM; NaCl, 150 mM; Tween20, 0.1%, pH 7.5) for 1 hr at room temperature and then incubated with monoclonal antibodies 4G8/6E10 diluted 1:5000 for 1 hr. The antigen–antibody complexes were detected by horseradish peroxidase–conjugated sheep anti-mouse immunoglobulin G (IgG) antibody at 1:5,000 dilution (GE Healthcare Bio-Sciences, NC) and visualized using SuperSignal (Pierce Chemical, IL) on X-Omat Blue XB-1 autoradiography film (Eastman Kodak Company, New Haven, CT). Autoradiographs were digitized and subjected to densitometric analysis using ImageJ software v1.34 (NIH, Bethesda, MD).

### Measurement of levels of plasma cholesterol

Plasma cholesterol level was measured by using a Cholesterol E kit (Wako Diagnostics, Richmond, VA)^45^. The Cholesterol E is an enzymatic colorimetric method for the quantitative determination of total cholesterol in the blood.

### Statistical Analysis

Object recognition data were analyzed by using paired, two tailed *t* test. Data from the radial arm maze and Thioflavin-T assay were analyzed by two-way repeated measures ANOVA. All other sensorimotor test, histological and biochemical measurements between groups were analyzed by using the unpaired Student’s *t* test with Welch’s correction following confirmation of normal data distribution by the Kolmogorov-Smirnov and Shapiro-Wilk tests. All statistical analyses were performed using GraphPad Prism 7.0 (Graphpad, San Diego, CA).

## Results

### Studies of CPO_Aβ17-21 P *in Vitro*

**Figure 1:**
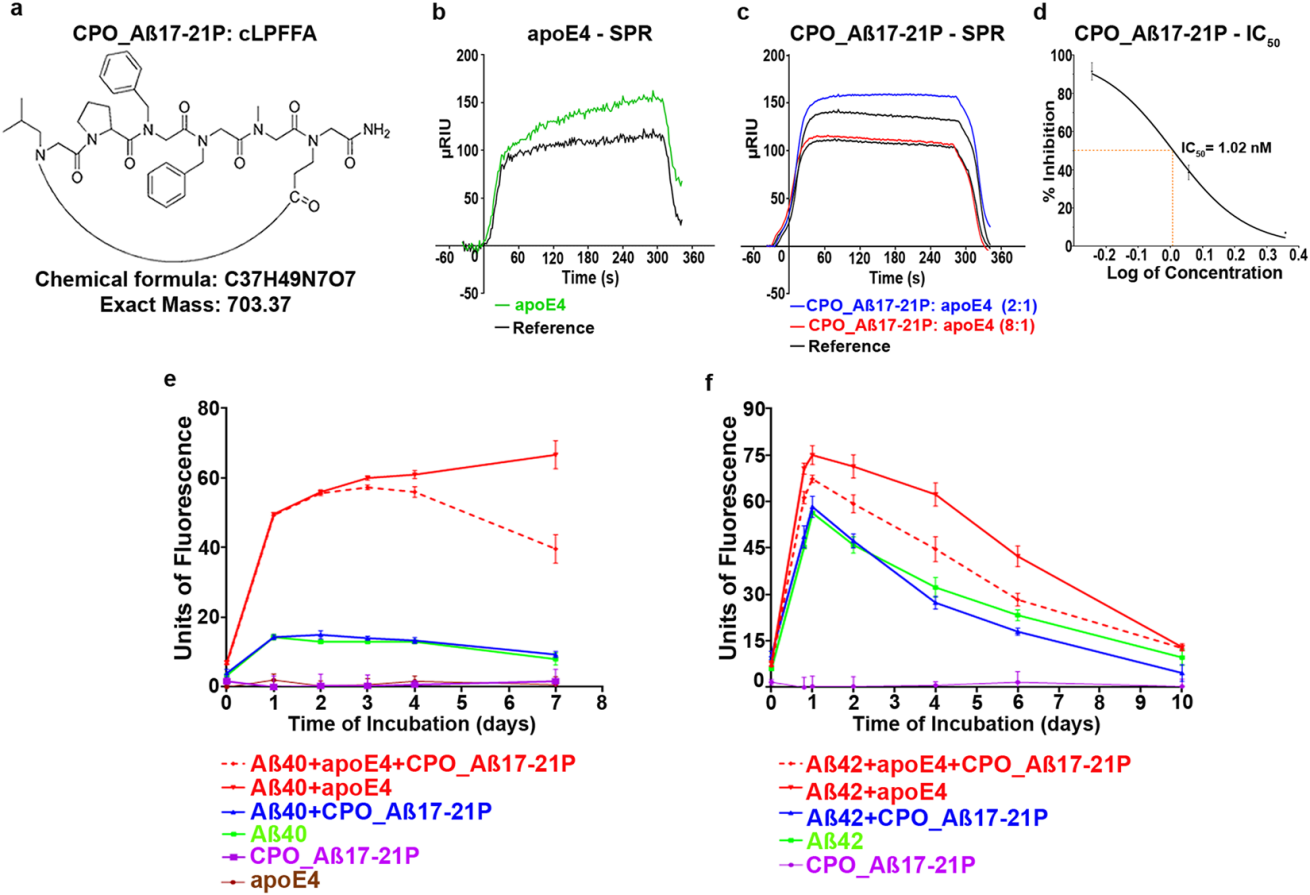
Molecular structure of peptoid CPO_A*β*17-21 P and its inhibitory effect on apoE4/A*β* 42 binding and reduction of apoE4 mediated A*β*40 and A*β*42 fibrillization. (**a**) Molecular structure of the peptoid CPO_A*β*17-21 P. (**b**) Surface Plasmon Resonance assay of apoE4/A*β*42 binding. Binding of lipidated apoE4 is shown by the green line with the black line representing the reference. (**c**) Surface Plasmon Resonance assay of CPO_A*β*17-21 P inhibition of apoE4/A*β*42 binding at a 2:1 (blue line, peptoid:apoE4) and 8:1 molar ratio (red line, peptoid:apoE4), with the black line below each colored line representing the corresponding reference. (**d**) Shown is the half-maximal inhibition (IC50) of lipidated apoE/A*β* binding by increasing concentrations of CPO_A*β*17-21 P derived from a one-site competition, nonlinear, regression equation of CPO_ Aβ17-21 P was 1.02 nM, calculated using GraphPad Prism 7.0 (Graphpad, San Diego, CA). (**e**) and (**f**) Thioflavin T A*β* aggregation assay, showing inhibition of apoE4 potentiated A*β*40 and A*β*42 fibril formation when incubated with CPO_A*β*17-21 P. Solid red line shows the A*β*40 (400 µM, in e) or A*β*42 (100 µM, in f) fibril formation increment when incubated with recombinant apoE4 at 100:1 molar ratio. Dashed red line shows reduction of fibril formation on A*β*40 (in (**e**) and A*β*42 (in **f**) when apoE4 was pre-incubated with peptoid CPO_A*β*17-21 P. Green line shows A*β*40 (in **e**) and A*β*42 (in **f**) alone, blue line indicates A*β*40 or A*β*42 incubated with peptoid, brown line indicates apoE4 alone and purple line shows peptoid CPO_A*β*17-21 P. Repeated measures analysis of variance followed by a Tukey post-hoc multiple comparison analysis showed *p* < 0.0001 for difference between group; A*β*40 + apoE4 + CPO_A*β*17-21 P versus A*β*40 + apoE4 *p* = 0.0034; A*β*42 + apoE4 + CPO_A*β*17-21 P versus A*β*42 + apoE4 *p* = 0.0011; no significant difference between A*β*40 + CPO_A*β*17-21 P and A*β*40 alone; no significant difference between A*β*42 + CPO_A*β*17-21 P and A*β*42 alone were found.

We designed 9 pairs of related linear and cyclic peptoids derived from A*β*12-28 P sequence to screen for new apoE/A*β* binding inhibitors with higher efficacy and safety (see Supplemental Figs 1 and 2 showing the structure of all tested peptoids). Surface Plasmon Resonance studies showed that three peptoid compounds CPO_A*β*17-21 P, CPO_A*β*17-21 and LPO_A*β*17-21 P inhibited apoE4/A*β*42 binding. CPO_Aβ17-21 P (Fig. 1a) produced the best inhibition of binding and was chosen as the lead peptoid for further testing. It has a molecular weight of 703.83 and a peptide-like backbone composed of only 5 peptoid units (Fig. 1a). There was a 50% inhibition of apoE4/A*β*42 binding (Fig. 1c) by the lead peptide, CPO_ A*β*17-21 P at a 2:1 molar ratio (peptoid:apoE4, blue line Fig. 1c) and a near complete inhibition at a 8:1 molar ratio (peptoid:apoE4, red line Fig. 1c). The half-maximal inhibition (IC_50_) derived from a one-site competition, nonlinear, regression equation of CPO_ A*β*17-21 P was 1.02 nM, calculated using GraphPad Prism 7.0 (Graphpad, San Diego, CA) (Fig. 1d), which is significantly better compared to our previously published IC_50_ (36.7 nM) of the A*β*17-28P^16^. Peptoids lack the hydrogen of secondary amides, and thus are unlikely to form *β*-sheet structures, which is associated with the toxicity of A*β* oligomers and A*β* derived peptides. Incubation of peptoid inhibitor CPO_A*β*17–21 P at 37 °C for 10days at concentrations up to 100 µM did not produce aggregates or fibrils, indicating that CPO_A*β*17–21 P is non-fibrillogenic *in vitro* (see purple line in Fig. 1e and f). Thioflavin T (ThT) aggregation assay of A*β40* and apoE4 protein pre-incubated with peptoid inhibitor CPO_A*β*17-21 P at a 1 to 2 molar ratio (apoE4: inhibitor), and of A*β*42 and apoE4 pre-incubated with peptoid inhibitor CPO_A*β*17-21 P at a 1 to 2 molar ratio (apoE4: inhibitor), showed a significant inhibition of apoE4 mediated A*β*40 (*p = *0.0034, by 2-way ANOVA followed by Tukey’s multiple comparisons test comparing the A*β*40 + apoE4 + CPO_A*β*17-21 P versus A*β*40 + apoE4 curves) or A*β*42 (*p* = 0.0011, by 2-way ANOVA followed by Tukey’s multiple comparisons test comparing the A*β*42 + apoE4 + CPO_Aβ17-21 P versus A*β*42 + apoE4 curves) *in vitro* aggregation (Fig. 1e and f). This degree of inhibition, by CPO_A*β*17–21 P, of A*β*40/42 aggregation enhancement by apoE4 was somewhat less compared to what we have previously reported with Aβ12-28 P^16^.

**Figure 2:**
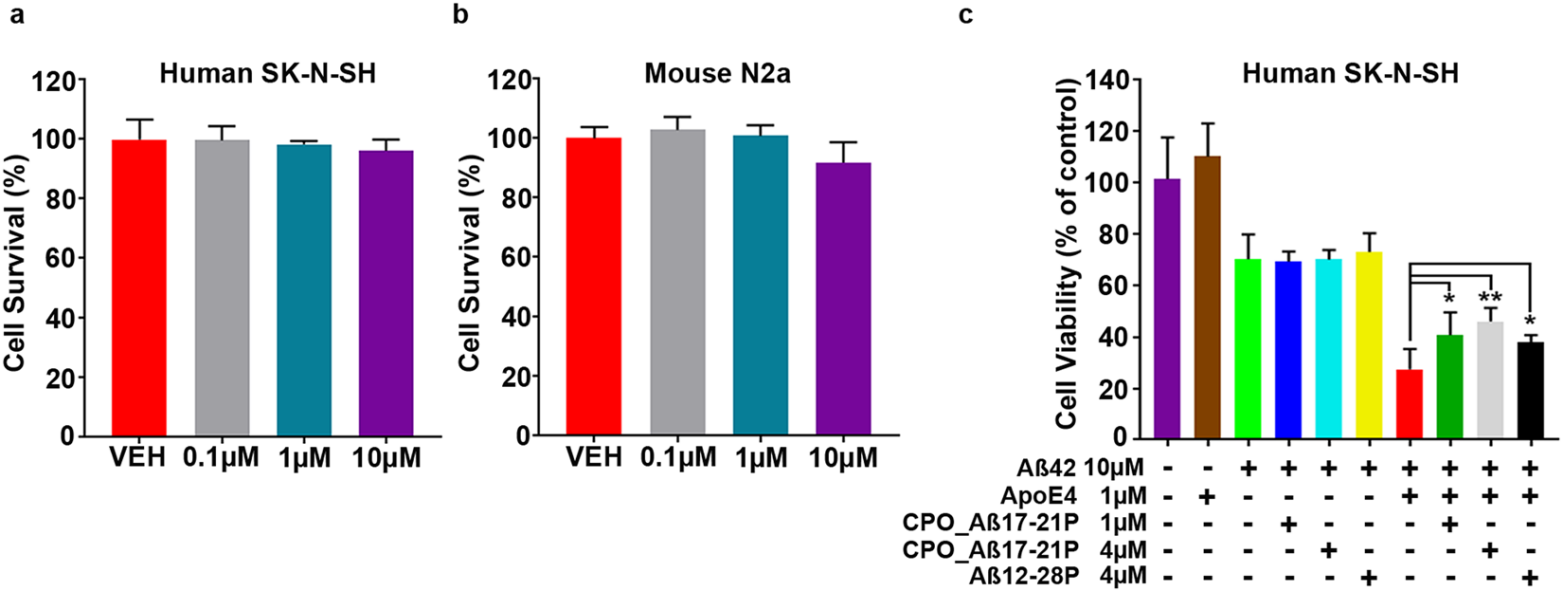
Cytotoxicity of peptoid CPO_ A*β*17-21 P on SK-N-SH human neuroblastoma cells and mouse N2a neuroblastoma cells and inhibition of A*β*42/apoE4 cytotoxicity by pre-incubation of apoE4 with CPO_A*β*17-21 P. (**a**) CellTiter-Blue® cytotoxicity assay showing no significant differences between vehicle and CPO_ A*β*17-21 P at 0.1, 1 and 10 µM. on human SK-N-SH neuroblastoma cells or on (**b**) mouse N2a neuroblastoma cells. (**c**) Toxicity effect of A*β*42 on human SK-N-SH neuroblastoma cells and potentiation of toxicity in presence of apoE4. CPO_ A*β*17-21 P peptoid reduces significantly the toxicity of A*β*42/apoE4 at 1 and 4 µM (**p < 0.01, *p < 0.05).

CPO_AB17-21P cytotoxicity was tested by MTS cell viability assays in human (SK-N-SH) and in mouse (N2a) neuroblastoma cell models. CPO_A*β*17–21 P is nontoxic to cells treated for 24 hrs at concentrations of 0.1 µM to 10 µM (Fig. 2a and b). In human SK-N-SH neuroblastoma cells, the apoE4-enhanced cytotoxicity of A*β*42 is neutralized by pre-incubation of apoE4 with binding inhibitors CPO_A*β*17–21 P or A*β*12-28 P (Fig. 2c). Under the same condition, CPO_A*β*17–21 P (**p < 0.01) has a greater apoE4 neutralization effect compared to A*β*12-28 P (*p < 0.05). A higher ratio of CPO_A*β*17–21 P to apoE4 pre-incubation treatment was associated with enhanced rescue effects on SK-N-SH cells, indicating greater apoE4 inhibition, which mirrors the SPR results.

### Studies of CPO_Aβ17-21 P in APP/PS1 AD Model Mice

#### Behavioral studies

**Figure 3:**
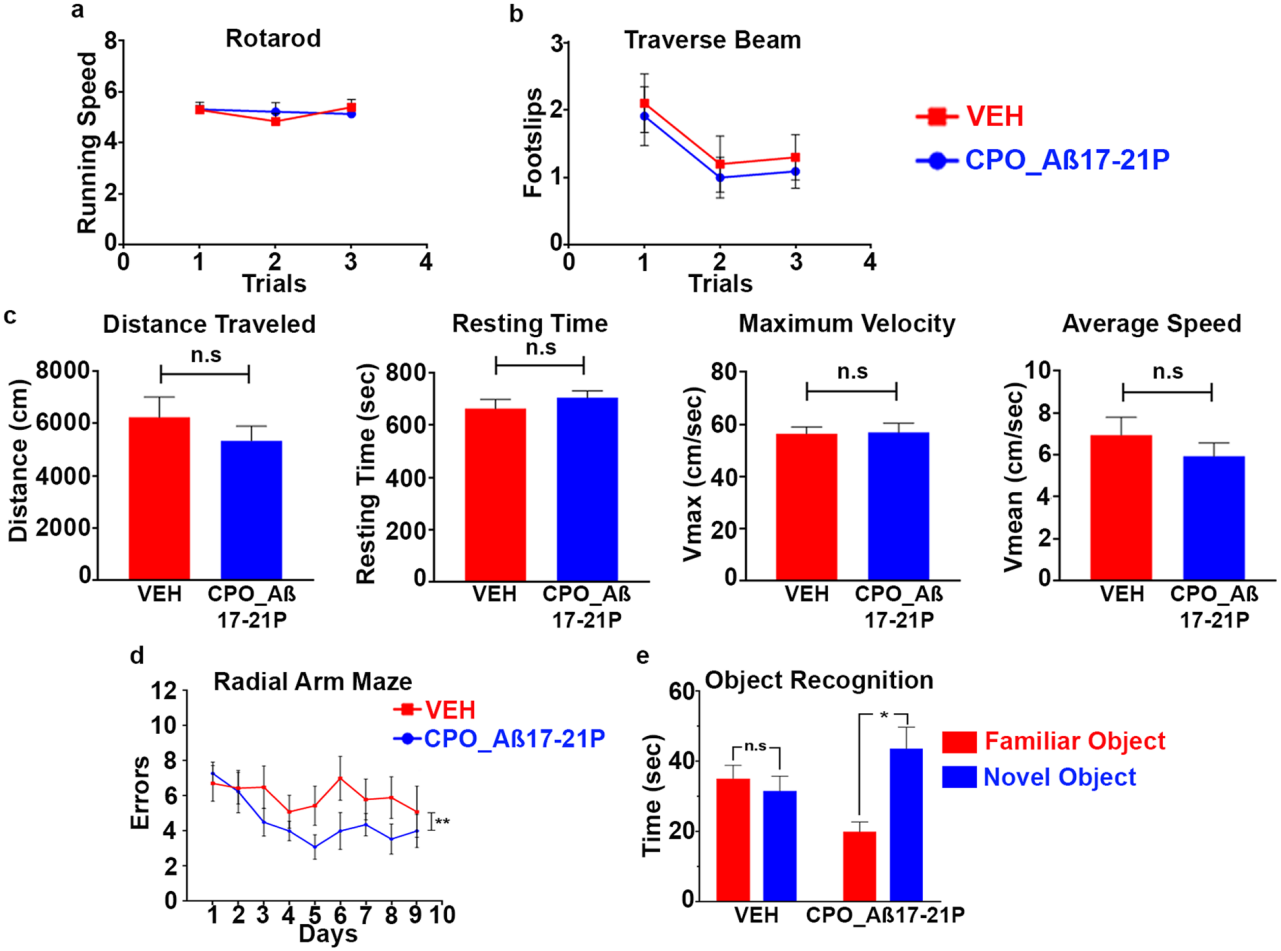
Cognitive testing and locomotor assessment of APP/PS1 mice treated with peptoid CPO_ A*β*17-21 P. (**a**) Rotarod and (**b**) Traverse beam locomotor tests showing no significant differences between vehicle group and mice treated with the peptoid (*p* > 0.05). (**c**) Open field locomotor test results showing no significant differences in distance traveled, resting time, maximum velocity and average speed between vehicle group and peptoid treated mice (p > 0.05, two-tailed unpaired *t-*test). (**d**) Radial arm maze. Number of errors plotted versus number of days. Significant differences between vehicle mice group and mice treated with the peptoid (**p < 0.01, by two-way ANOVA for treatment effect). (**e**) Object recognition test showing the average time spent with the familiar and novel objects. Significant differences in the time that treated mice spent with the novel object compared to the vehicle group (*p < 0.05, two-tailed paired *t*-test). There was no significant difference between the time spent with the familiar and novel objects in the vehicle control group.

The APP/PS1 AD mouse is a model of cerebral amyloidosis, that rapidly develops amyloid associated pathologies with a robust gliosis and thus facilitates a quick readout of therapeutic effects. To test the *in vivo* therapeutic effects of the lead peptoid, CPO_ A*β*17-21 P, we systematically treated APP/PS1 AD mice twice a week for four months with 0.20 mg of CPO_A*β*17-21 P per mouse (n = 11, 5 male, 6 female) or with vehicle saline (n = 10, 5 male, 5 female) as a control. Sensorimotor and cognitive testing of the APP/PS1 AD mice receiving CPO_AB17-21P or vehicle revealed no observable differences or anomalies in rotarod and locomotor activities (Fig. 3a–c). APP/PS1 AD mice treated with CPO_A*β*17–21 P showed a significant cognitive improvement as measured by radial arm maze (Fig. 3d; **p < 0.01 by two-way ANOVA for treatment effect) and novel object recognition (Fig. 3e; *p = 0.016), two standard cognitive tests. There was no significant difference between APP/PS1 AD mice treated with CPO_Aβ17–21 P and controls in latency measurements during the radial arm maze testing by two-way ANOVA (p = 0.09 for treatment effect, p = 0.0018 for days effect; data not shown).

#### Biochemical and immunohistochemical studies

**Figure 4:**
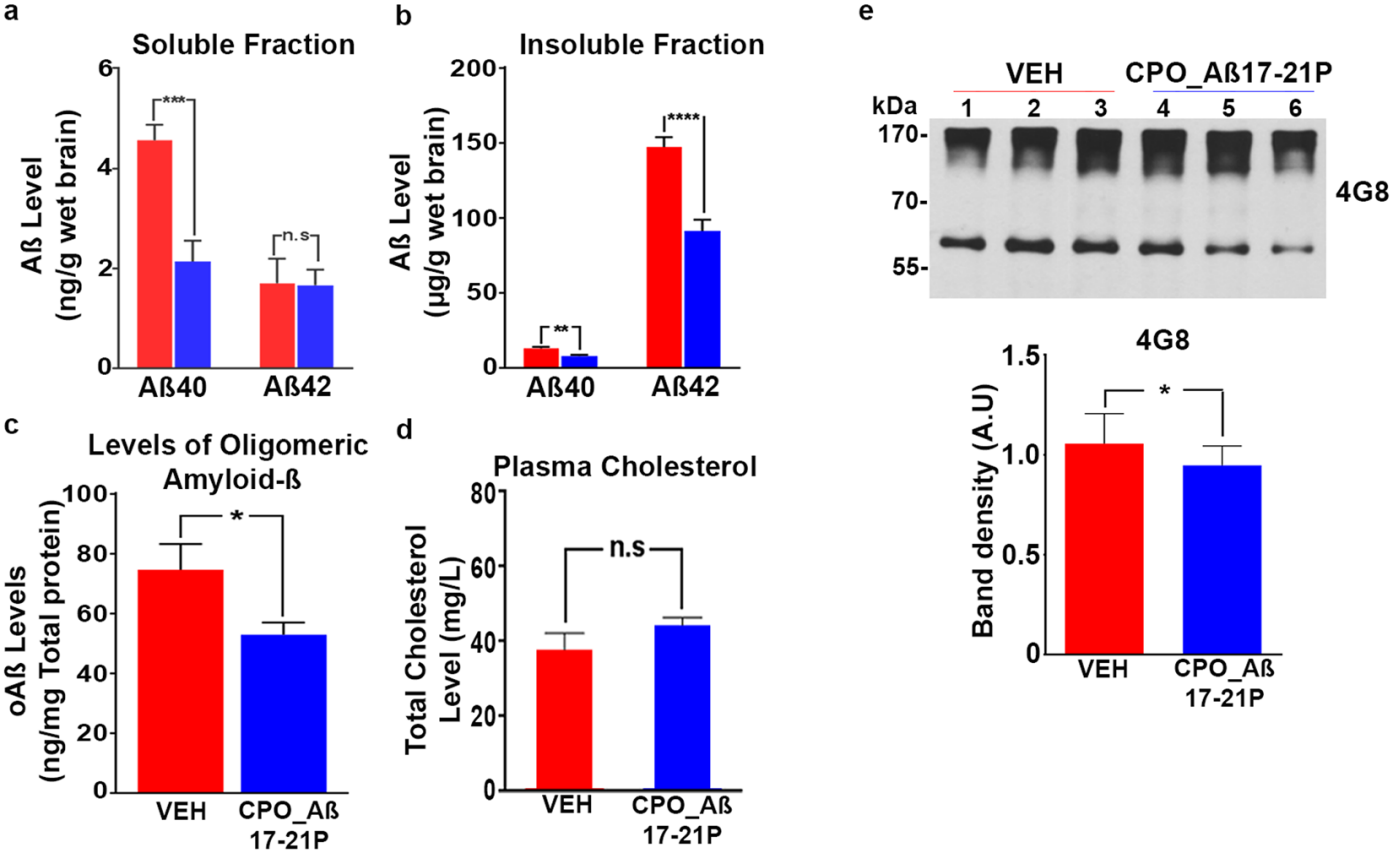
Reduction of soluble and insoluble Amyloid-*β* levels on APP/PS1 mice treated with peptoid CPO_ A*β*17-21 P. (**a**) Reduction of A*β*40 in the soluble fraction extracted with diethylamine (DEA) from APP/PS1 mice brain homogenates (***p < 0.001, two-tailed unpaired t-test). (**b**) Reduction of A*β*40 and A*β*42 levels in the insoluble fraction extracted with formic acid (FA) (***p < 0.001 and ****p < 0.0001 respectively, two-tailed unpaired t-test). (**c**) Reduction of A*β* oligomeric species measured by ELISA assay (*p < 0.05, two-tailed unpaired t-test). (**d**) Plasma cholesterol levels on APP/PS1 mice treated with peptoid CPO_ A*β*17-21 P (*p* > 0.05, two-tailed unpaired *t*-test). (**e**) Reduction of A*β* oligomeric species shown by western blot with anti-amyloid antibody 4G8 on APP/PS1 mice brain homogenates (*p < 0.05, two-tailed unpaired *t*-test).

**Figure 5:**
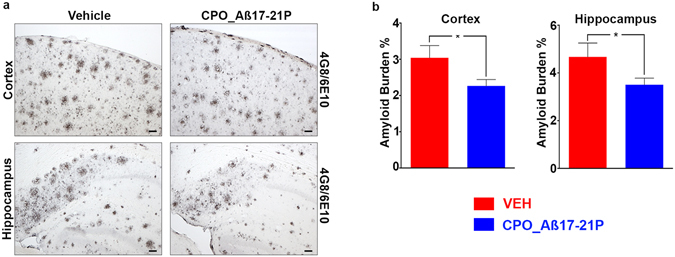
Amyloid-*β* plaque burden on brains of APP/PS1 mice treated with peptoid CPO_ A*β*17-21 P or vehicle alone. (**a**) Treatment with CPO_ A*β*17-21 P decreased cortical and hippocampal burden in APP/PSI mice. Scale bars represent 100 μm. (**b**) There was a significant reduction observed in 6E10/4G8 immunoreactivity in both cortex and hippocampus in the CPO_ A*β*17-21 P treated mice versus vehicle treated mice, *p < 0.05, one-tailed unpaired *t*-test.

This cognitive improvement was associated with reduction of insoluble and soluble A*β* peptide/oligomer levels in the brain (Fig. 4a and b), as well as reduction of total amyloid burden assessed by immunohistochemical analysis in both the cortex (*p* = 0.0226) and the hippocampus (*p* = 0.0361) (Fig. 5). Significantly, treatment with CPO_Aβ17-21 P reduced oligomer levels, the species most linked to neuronal toxicity^46^ (Fig. 4c and e). A potential risk of therapeutic approaches that target A*β* deposition involves elevated levels of soluble A*β* which may enhance formation of toxic oligomer species. We demonstrate that at least in this model, this is not the case. We also determined if CPO_A*β*17–21 P treatment effected plasma total cholesterol levels. There was no significant difference in the plasma total cholesterol level between vehicle and CPO_A*β*17–21 P treated mice (Fig. 4d).

**Figure 6:**
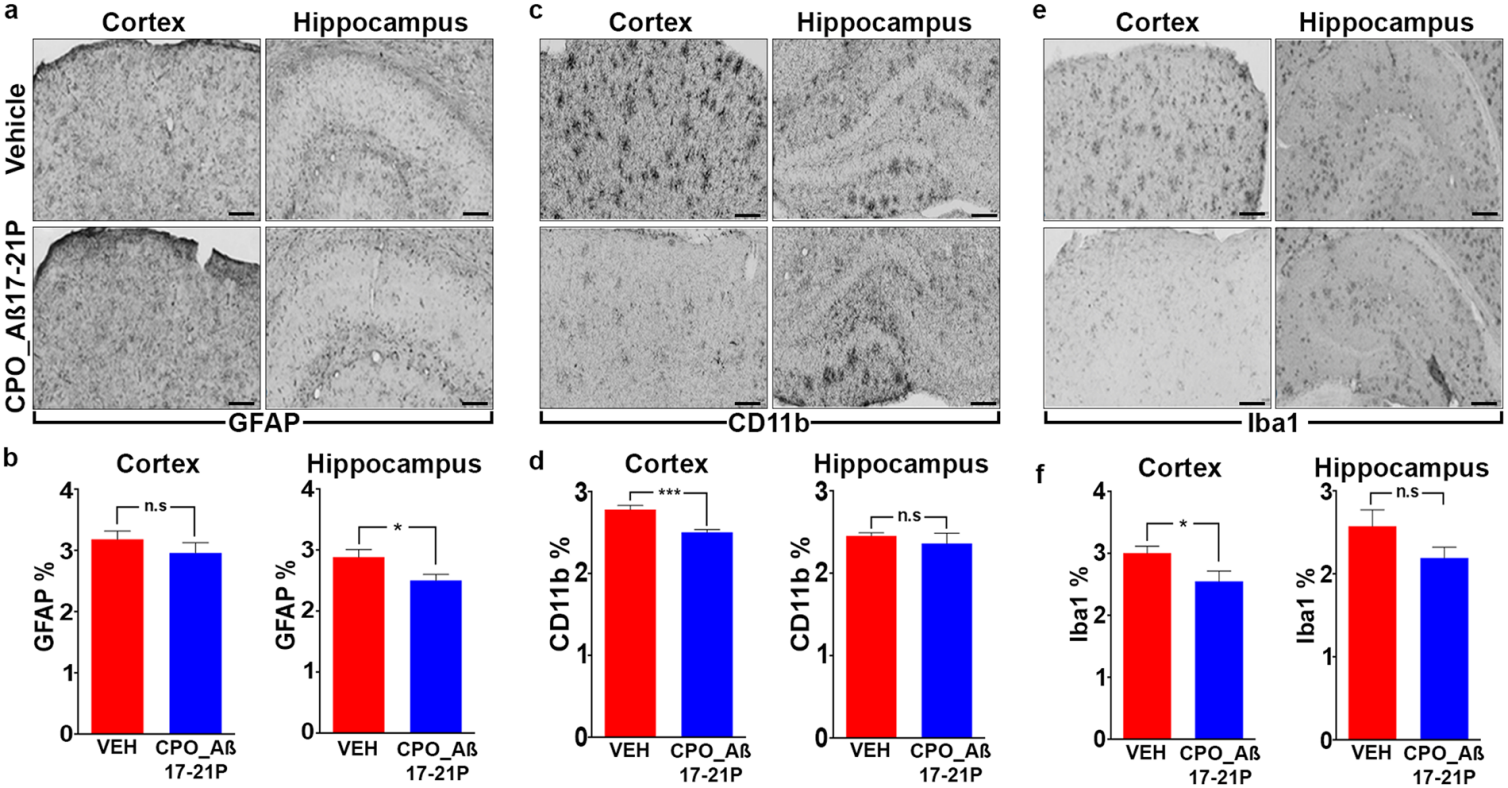
Levels of reactive astrocytes and microglia in brains from APP/PS1 mice treated with CPO_ A*β*17-21 P or vehicle. (**a**) Treatment with CPO_ A*β*17-21 P had no significant effect on cortical astrogliosis, but it was associated with decreased hippocampal astrogliosis in APP/PS1 mice, (**b**) as quantified by GFAP immunoreactivity. (**c**) Treatment with CPO_ A*β*17-21 P decreased cortical, but had no significant effect on hippocampal microgliosis in APP/PS1 mice, (**d**) as quantified by CD11b immunoreactivity, (**e**) CPO_ A*β*17-21 P treatment reduced microgliosis in the cortex, but had no significant effect on hippocampal microgliosis of APP/PS1 mice, (**f**) as quantified by Iba1 immunoreactivity, (Scale bars represent 100 μm.*p < 0.05, ***p < 0.001, one-tailed unpaired *t-*test).

Another potential risk in targeting A*β* deposition is exacerbated brain inflammation with resultant neuronal dysfunction/death. Our studies document that immunoreactivity of GFAP (Fig. 6a and b), CD11b (Fig. 6c and d) and Iba1 (Fig. 6e and f), biomarkers for microgliosis (Iba1 and CD11b) and astrogliosis (GFAP), are unchanged (with a trend for reduction) or are significantly reduced in the CPO_A*β*17-21 P treated Tg mice versus vehicle mice (Fig. 6).

## Discussion

The apoE/Aβ interaction plays a key role in the conformational transformation of soluble A*β* and A*β* deposits in AD brains^3, 5, 47, 48^. ApoE has multiple important normal functions in the brain, being the major CNS cholesterol and other lipid carrier, as well as being involved in synaptic plasticity, glucose metabolism, mitochondrial function, and vascular integrity^4, 48^. In AD apoE influences both the aggregation state and clearance of Aβ in an isotype specific manner^3, 5, 48–50^. ApoE has been shown to enhance aggregation of Aβ with apoE4 > apoE3 > apoE2^8, 9, 33, 51^, as well having effects on the stabilization of Aβ oligomers with apoE4 having the greatest impact^52, 53^. Under physiological conditions, relatively little normal, soluble Aβ (sAβ) is bound to apoE^15^, with the major CNS Aβ binding protein being apoJ^54, 55^. However, in AD with the shift in the aggregate state of Aβ, there is more interaction with apoE^12, 50, 56^. It has also been shown that apoE4 is less effective at clearance of Aβ compared to apoE3^57^. Hence it might be suggested that a strategy of blocking the binding between apoE and Aβ might promote Aβ deposition, as it would inhibit clearance. Pivotal *in vivo* studies have shown that this is not the case. Knock out of apoE greatly reduces fibrillar amyloid deposition^58^, with apoE4 expressing AD transgenic mice have greater amyloid deposition compared to apoE3 or E2 expressing mice^59, 60^. Other Aβ binding proteins such as apoJ or α2-macroglobulin are associated with pathways that are much more effective at Aβ clearance compared to clearance of Aβ/apoE complexes^3, 49, 50, 61^. Hence the net effect of blocking the Aβ/apoE interaction is to inhibit deposition and enhance clearance. In addition, this strategy has the benefit of not interfering with the many normal and beneficial functions of apoE. In prior studies we showed that treatment with A*β* 12-28 P, a peptide homologous to the specific apoE binding domain of A*β* in two AD Tg mouse models with primarily amyloid plaque deposition and in one AD model with primarily CAA resulted in a significant reduction of A*β* burden in both brain parenchyma and in brain vessels compared to age matched vehicle-treated Tg mice^16–18^. We also demonstrated that blocking the apoE/A*β* interaction with A*β*12-28 P in triple transgenic mice ameliorates AD related A*β* and tau pathology^19^. A*β*12-28 P treatment in an amyloid mouse model with apoE2-TR or apoE4-TR mouse background resulted in A*β* oligomer and plaque load reduction and alleviated neuritic degeneration indicating that inhibition of A*β*/apoE interaction appears to effectively block aggregation and deposition of A*β*, regardless of apoE isoform^20^.

In our current study to advance our novel approach towards clinical application, we designed 9 pairs of related linear and cyclic peptoid compounds derived from the A*β*12-28 P sequence to screen for new apoE/A*β* binding inhibitors with a potentially higher efficacy and safety. The lead peptoid screened by SPR, CPO_A*β*17-21 P decreased the apoE4/A*β*42 binding at a 2:1 molar ratio (peptoid:apoE4) and almost completely blocked binding at a 8:1 molar ratio (peptoid:apoE4). Significantly, the half-maximal inhibition (IC_50_) derived from a one-site competition, nonlinear, regression equation of CPO_A*β*17-21 P was 1.02 nM compared to 36.7 nM for the model peptide, A*β*12-28P^16^. Previous studies have shown that the critical region for A*β* binding to apoE corresponds to residues 17-21 with the lysine at residue 16 being particularly important^62, 63^. Hence it is not surprising that a peptoid corresponding to this sequence would be an effective inhibitor of the A*β*/apoE interaction.

We show this peptoid is non-fibrillar and Thioflavin T aggregation assay testing showed a significant inhibition of apoE mediated aggregation of A*β*. CPO_ A*β*17-21 P was not toxic in mouse N2a and human SK-N-SH neuroblastoma cell lines. Moreover CPO_ A*β*17-21 P is a more potent inhibitor of apoE-enhanced A*β* 42 cytotoxicity than A*β*12-28 P in human SK-N-SH neuroblastoma cells. APP/PS1 AD mice treated with CPO_ A*β*17-21 P had a significant cognitive improvement, reduction of soluble and insoluble A*β* peptide/oligomer levels in brain and lower total amyloid burden in cortex and hippocampus. Importantly, CPO_ A*β*17–21 P treatment ameliorates A*β* related pathology and cognitive deficit using a 7.5 fold lower dose (0.2 mg per mouse, twice per week) compared to the A*β*12-28 P treatment dose we previously used on 3xTg mice (1 mg per mice, three times per week)^19^. This argues that the new peptoid inhibitor CPO_A*β*17–21 P, with a very low molecular weight (<1 kDa) and inherently protease resistant compound, has better bioavailability/biostability compared to A*β*12-28 P.

A potential risk inherent in targeting A*β* deposition is increasing the pool of soluble A*β* which may potentiate formation of the toxic oligomer species, as has been shown for some immunotherapeutic approaches^64^. Although apoE has a dual role in A*β* deposition and clearance, CPO_A*β*17-21 P inhibition of apoE4/A*β*42 interaction in APP/PS1 AD mice did not affect the soluble A*β* pool. Brain inflammation is another potential risk when targeting A*β* deposition. Our studies show, that Iba1 and CD11b (both markers for microgliosis), and GFAP (a biomarker for astrogliosis) immunoreactivity is reduced or unchanged in the CPO_A*β*17-21 P treated Tg mice.

Currently, there is no effective therapy for AD, a highly prevalent, devastating neurodegenerative disease expected to increase in the future with the aging population^1, 65^. Our novel therapy of blocking apoE/A*β* interaction has ameliorated all the pathological features tested in the present study: improved memory deficits, reduction of amyloid burden and tau pathology and reduction of vascular amyloid deposition. A study which used A*β*12-28 P to block apoE/A*β* interaction in an amyloid mouse model with apoE2-TR or apoE4-TR mouse background resulted in A*β* plaque load and oligomer reduction and ameliorated neuritic degeneration^20^. Thus this therapy is not apoE isoform restrictive. This approach does not preclude concomitant therapies which may have a synergistic effect that procures an effective treatment. Our current study provides further evidence that targeting the apoE/A*β* interaction is an attractive and viable therapeutic approach^3, 7^.

## Electronic supplementary material

Supplementry Information
