# Supplementary material for: Targeting Apolipoprotein E/Amyloid β Binding by Peptoid CPO_Aβ17-21 P Ameliorates Alzheimer’s Disease Related Pathology and Cognitive Decline
Source: Sci Rep. 2017 Aug 14;7:8009. doi: 10.1038/s41598-017-08604-8 (PMC5556019; doi:10.1038/s41598-017-08604-8)
Supplement: Supplementary file 1 — Supplementry Information [file 41598_2017_8604_MOESM1_ESM.pdf]

Supplemental Information for

Targeting Apolipoprotein E/Amyloid β Binding by Peptoid CPO_Aβ17-21P Ameliorates Alzheimer's Disease Related Pathology and Cognitive Decline

Shan Liu[1], Shinae Park[2], Grant Allington[3], Frances Prelli[1], Yanjie Sun[1], Mitchell Martá-Ariza[1], Henrieta Scholtzova[1], Goutam Biswas[2], Bernard Brown[1], Philip B. Verghese[4], Pankaj D. Mehta[5], Yong-Uk Kwon[2*], Thomas Wisniewski[6*]

[1]Center for Cognitive Neurology, Department of Neurology, New York University School of Medicine
[2]Department of Chemistry and Nanoscience, Ewha Womans University, Korea
[3]Department of Chemistry, New York University
[4]C2N Diagnostics, Center for Emerging Technologies, 4041 Forest Park Avenue, St. Louis, MO 63108
[5]Department of Immunology, New York State Institute for Basic Research in Developmental Disabilities
[6]Center for Cognitive Neurology, Departments of Neurology, Psychiatry and Pathology, Neuroscience Institute, New York University School of Medicine

*co-corresponding authors:
Thomas Wisniewski
New York University School of Medicine
Alexandria ERSP, 450 East 29th Street
NYU School of Medicine, New York, NY, 10016 USA
e-mail: Thomas.wisniewski@nyumc.org

Yong-Uk Kwon
Department of Chemistry and Nanoscience,
Ewha Womans University,
Seoul 03760, Korea.
E-mail: yukwon@ewha.ac.kr

LPO_Aβ17-21: LVFFA

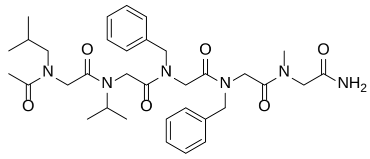

LPO_Aβ17-21P: LPFFA

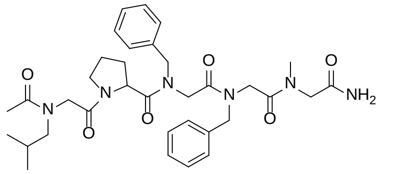

LPO_Aβ15-24: QKLVFFAEDV

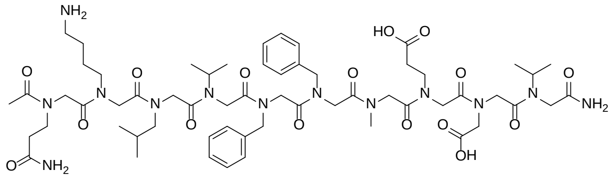

LPO_Aβ15-24P: QKLPFFAEDV

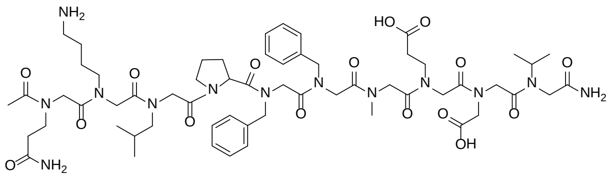

LPO_Aβ17-21VV: GLVFFAGSGLVFFAG

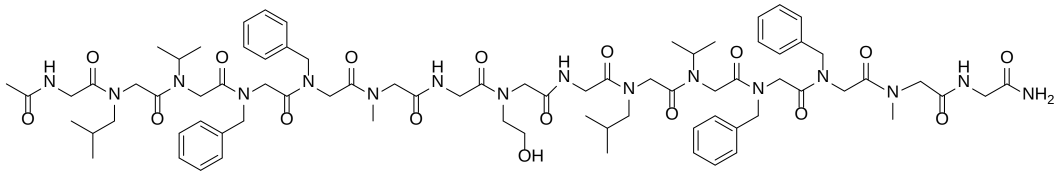

LPO_Aβ17-21VP: GLVFFAGSGLPFFAG

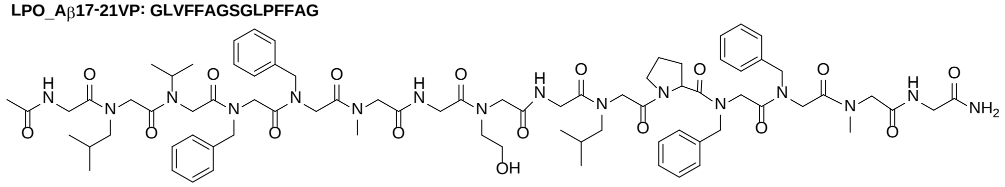

LPO_Aβ17-21PV: GLPFFAGSGLVFFAG

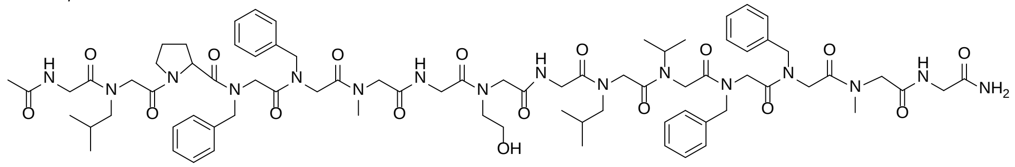

LPO_Aβ17-21PP: GLPFFAGSGLPFFAG

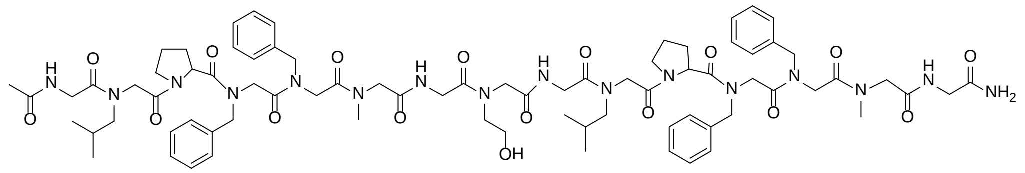

LPO_Aβ12-28P: VHHQKLPFFAEDVGSNK

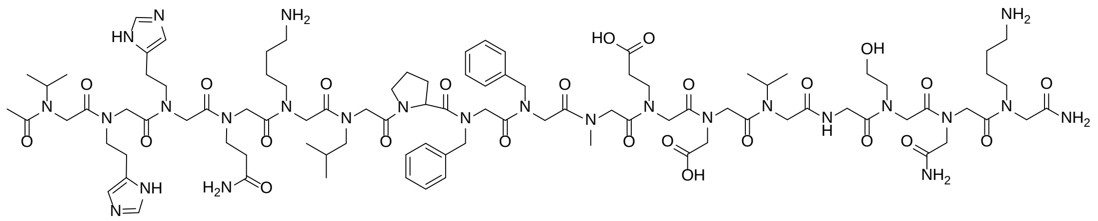

Supplemental Figure 1

CPO_Aβ17-21: cLVFFA

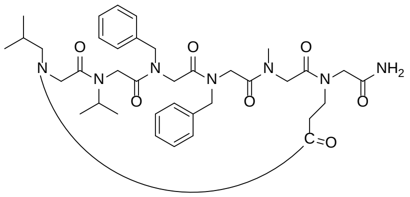

CPO_Aβ17-21P: cLPFFA

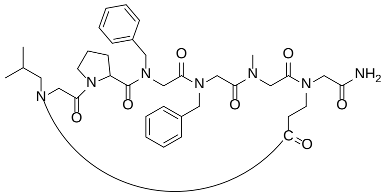

CPO_Aβ15-24: cQKLVFFAEDV

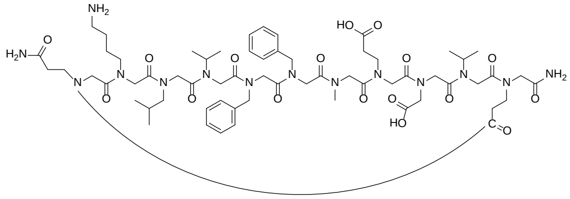

CPO_Aβ15-24P: cQKLPFFAEDV

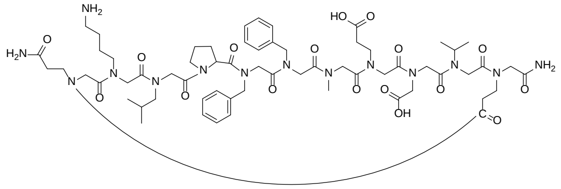

CPO_Aβ17-21VV: cGLVFFAGSGLVFFAG

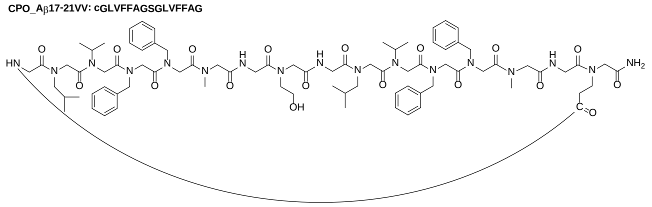

CPO_Aβ17-21VP: cGLVFFAGSGLPFFAG

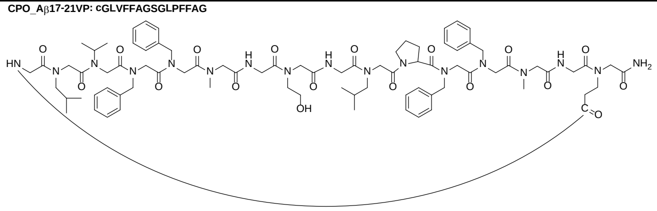

CPO_Aβ17-21PV: cGLPFFAGSGLVFFAG

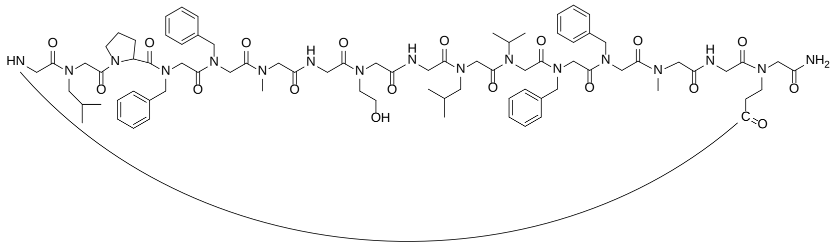

CPO_Aβ17-21PP: cGLPFFAGSGLPFFAG

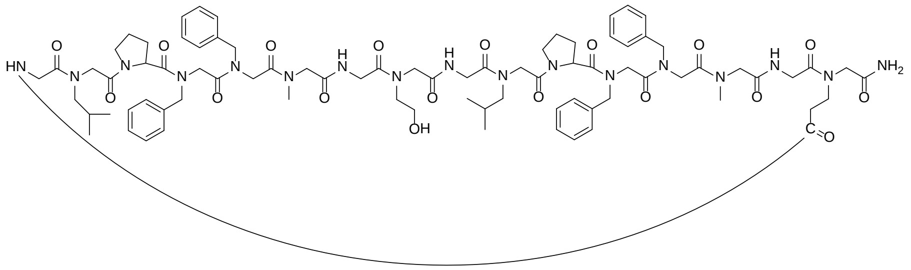

CPO_Aβ12-28P: cVHHQKLPFFAEDVGSNK

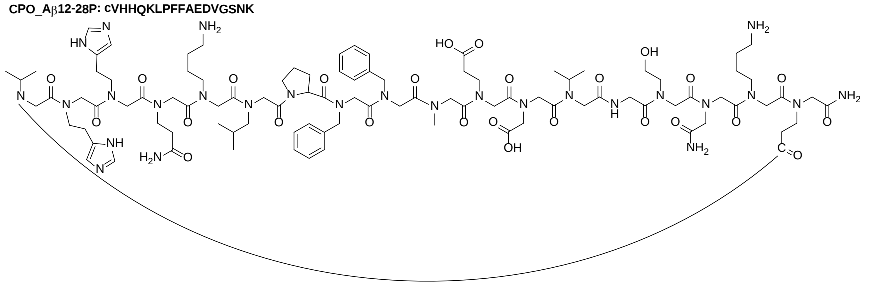

Supplemental Figure 2
